# Produkte mit Mikronährstoffen: Arzneimittel oder Lebensmittel?

**DOI:** 10.1007/s00103-025-04120-7

**Published:** 2025-09-19

**Authors:** Hendrik Schulze, Benjamin Conrads, Ulrike Saerbeck, Evelyn Breitweg-Lehmann

**Affiliations:** Abteilung 1, Referat 111 „Grundsatzangelegenheiten, Lebensmittel“, Bundesamt für Verbraucherschutz und Lebensmittelsicherheit (BVL), Gerichtstraße 49, 13347 Berlin, Deutschland

**Keywords:** Lebensmittelsicherheit, Nahrungsergänzungsmittel, Funktionsarzneimittel, Pharmakologische Wirkung, Abgrenzung, Food safety, Food supplements, Functional medicinal product, Pharmalogical effect, Demarcation

## Abstract

Nahrungsergänzungsmittel spielen eine Rolle im Alltag vieler Verbraucher, deren Wunsch nach einem gesunden Leben durch einen wachsenden Markt aus verschiedenartigen Präparaten vermeintlich erfüllt wird. Obwohl sie in der Aufmachung häufig Arzneimitteln ähneln, sind Nahrungsergänzungsmittel auch Lebensmittel, für die die gleichen rechtlichen Bestimmungen wie für andere Lebensmittel gelten. Die Abgrenzung zwischen Arzneimitteln und Lebensmitteln – insbesondere Nahrungsergänzungsmitteln – stellt jedoch eine komplexe rechtliche und praktische Herausforderung dar. Beide Produktgruppen können sich in Zusammensetzung, Darreichungsform und Wirkung stark ähneln, unterscheiden sich jedoch grundlegend in den regulatorischen Anforderungen. Dem Inverkehrbringen von Arzneimitteln geht ein strenges Zulassungsverfahren mit Wirksamkeits‑, Sicherheits- und Qualitätsnachweisen voraus. Im Gegensatz dazu gibt es für Nahrungsergänzungsmittel, die oft identische Stoffe in niedrigeren Dosierungen enthalten, lediglich ein Anzeigeverfahren ohne vorausgehende Prüfung. Zum Teil werden Erzeugnisse fälschlicherweise als Nahrungsergänzungsmittel in Verkehr gebracht, obwohl sie aufgrund ihrer Wirkung oder Aufmachung als Arzneimittel einzustufen sind. Die Einstufung als Arzneimittel erfolgt stets in einer Einzelfallprüfung anhand verschiedener Faktoren, wobei die nennenswerte pharmakologische Wirkung in der Regel das zentrale Kriterium darstellt. Trotz des dynamischen und vielfältigen Marktes gelingt es den Überwachungsbehörden, derartige Produkte rechtssicher und im Sinne des Verbraucherschutzes einzustufen.

## Abstract (en)

Food supplements play an important role in the everyday lives of many consumers, whose desire for a healthy lifestyle is supposedly fulfilled by a growing market of a variety of preparations. Although they often resemble medicinal products in their presentation, food supplements are also foodstuffs and are subject to the same legal provisions as other foodstuffs. However, the demarcation between medicinal products and foodstuffs—especially food supplements—poses a complex regulatory and practical challenge. Both product groups can be similar in terms of composition, dosage form, and effect, but they differ fundamentally in their regulatory requirements. Placing medicinal products on the market is preceded by a strict authorization procedure with proof of efficacy, safety and quality. In contrast, food supplements, which often contain identical substances in lower dosages, are subject only to a notification procedure without prior authorisation. In some cases, products are incorrectly marketed as food supplements even though they should be classified as medicinal products due to their effects or presentation. The classification as a medicinal product is always based on a case-by-case assessment of various factors, with the pharmacological effect usually being the key criterion. Despite the dynamic and diverse market, however, the authorities succeed in classifying such products in a legally compliant manner and in the interests of consumer protection.

## Einleitung

Die externe Zufuhr von Mikronährstoffen ist Voraussetzung für eine Vielzahl von essenziellen Körperfunktionen, wie z. B. für den Knochenaufbau, das Immun- und das Nervensystem. Im Gegensatz zu den Makronährstoffen wie Kohlenhydrate, Fette und Proteine braucht der Körper Mikronährstoffe nur in geringen, jedoch lebensnotwendigen Mengen. Der Begriff der Mikronährstoffe umfasst klassischerweise Vitamine und Mineralstoffe, einschließlich Spurenelemente. Das Streben nach einem gesundheitsorientierten Lebensstil erfährt seit Jahren einen deutlichen Aufschwung. Damit verbunden steigen auch das Angebot und die Verfügbarkeit von Produkten, die zur individuellen Gesundheitsförderung beworben werden. Allein im Jahr 2024 wurden 19.338 Nahrungsergänzungsmittel beim Bundesamt für Verbraucherschutz und Lebensmittelsicherheit (BVL) für das erstmalige Inverkehrbringen gemäß § 5 Absatz 1 Nahrungsergänzungsmittelverordnung (NemV) angezeigt [1, 2]. Auch wenn es immer wieder Trends für z. B. bestimmte pflanzliche Inhaltsstoffe gibt, sind die Mikronährstoffe die Dauerbrenner bei den Nahrungsergänzungsmitteln. Mikronährstoffe kommen als natürlicher Bestandteil in nahezu allen Lebensmitteln vor.

Grundsätzlich ist Deutschland laut Einschätzung der Deutschen Gesellschaft für Ernährung kein „Vitaminmangelland“ [3]. Die mediane Zufuhr der meisten Vitamine und Mineralstoffe liegt im Bereich der D‑A-CH-Referenzwerte [4]. Zur Prävention oder Behandlung von Nährstoffmangelerkrankungen können Arzneimittel ärztlich verschrieben werden. Mikronährstoffe kommen in allgemeinen Lebensmitteln vor und werden sowohl in Nahrungsergänzungsmitteln als auch in Arzneimitteln eingesetzt. Bei vielen Erzeugnissen, die als Nahrungsergänzungsmittel in Verkehr gebracht werden, stellt sich deshalb die Frage, ob es sich um ein Lebensmittel oder nicht doch um ein Arzneimittel handelt. Primär verantwortlich für die korrekte Einstufung als Lebensmittel ist der Lebensmittelunternehmer. Bei diesen sogenannten Borderline-Produkten stellt die korrekte Einstufung sowohl für die Hersteller als auch die Überwachungsbehörden häufig eine Herausforderung mit vielen zu betrachtenden Faktoren dar. Vom Ergebnis der Prüfung hängen die Anwendung unterschiedlicher Rechtsgrundlagen, Zuständigkeiten und schlussendlich auch die Marktzugangsverfahren der Erzeugnisse ab.

Dieser Beitrag behandelt die rechtlichen Grundlagen und praktischen Herausforderungen bei der Abgrenzung von Borderline-Produkten. Dabei werden neben dem fachlichen Vorgehen unter Berücksichtigung der aktuellen Rechtsprechung auch der Aufbau der Überwachung sowie nationale und internationale Initiativen zur Einstufungshilfe vorgestellt und in einem Fazit zusammengeführt.

### Grundlagen für die Abgrenzung zwischen Lebensmitteln und Arzneimitteln

Das Vorgehen bei der Abgrenzung von Nahrungsergänzungsmitteln und Arzneimitteln ergibt sich grundlegend aus den Begriffsdefinitionen der unterschiedlichen Rechtsgrundlagen sowie den darauf aufbauenden maßgeblichen Gerichtsentscheidungen. Gemäß Artikel 2 Satz 1 Verordnung (EG) Nr. 178/2002 [5] sind „Lebensmittel alle Stoffe oder Erzeugnisse, die dazu bestimmt sind oder von denen nach vernünftigem Ermessen erwartet werden kann, dass sie in verarbeitetem, teilweise verarbeitetem oder unverarbeitetem Zustand von Menschen aufgenommen werden“. Da diese Beschreibung auch auf klassische Arzneimittel zutrifft, schließt Artikel 2 Satz 3 Buchstabe d) Verordnung (EG) Nr. 178/2002 Arzneimittel explizit von der Definition aus. In gleicher Weise schließt der Arzneimittelbegriff in § 2 Absatz 3 Nr. 2 Arzneimittelgesetz (AMG [6]) auch Lebensmittel aus. Darüber hinaus wird durch die sogenannte Zweifelsregelung (syn. Zweifelsfallregelung) gemäß § 2 Absatz 3a AMG bestimmt, dass ein Erzeugnis, das unter Berücksichtigung aller seiner Eigenschaften sowohl unter die Definition eines Arzneimittels als auch unter z. B. die Definition eines Lebensmittels fallen kann, ein Arzneimittel ist. Dabei ist zu beachten, dass hierdurch keine Zweifel an der Arzneimitteleigenschaft überwunden werden können [7], sondern dass der Nachweis der Arzneimitteleigenschaft vor der Anwendung der Zweifelsregelung uneingeschränkt geführt werden muss [8].

#### Definitionen

Gemäß § 1 Absatz 1 NemV handelt es sich bei Nahrungsergänzungsmitteln um ein „Lebensmittel, das

1. dazu bestimmt ist, die allgemeine Ernährung zu ergänzen,
2. ein Konzentrat von Nährstoffen oder sonstigen Stoffen mit ernährungsspezifischer oder physiologischer Wirkung allein oder in Zusammensetzung darstellt und
3. in dosierter Form, insbesondere in Form von Kapseln, Pastillen, Tabletten, Pillen und anderen ähnlichen Darreichungsformen, Pulverbeuteln, Flüssigampullen, Flaschen mit Tropfeinsätzen und ähnlichen Darreichungsformen von Flüssigkeiten und Pulvern zur Aufnahme in abgemessenen kleinen Mengen, in den Verkehr gebracht wird.“

Bei Nährstoffen handelt es sich nach § 1 Absatz 2 NemV definitionsgemäß um Vitamine und Mineralstoffe, einschließlich Spurenelementen. In § 3 NemV wird weiter definiert, dass „nur die in Anhang I der Richtlinie 2002/46/EG […] aufgeführten Nährstoffe im Sinne des § 1 Absatz 2 in den in Anhang II der Richtlinie 2002/46/EG aufgeführten Formen verwendet werden dürfen“.

Bei den drei genannten Hauptmerkmalen spielt die dosierte Form aufgrund der offensichtlichen Überschneidung mit Arzneimitteln eine eher untergeordnete Rolle in der Abgrenzung.

Die europäische Richtlinie 2001/83/EG [9] umsetzend wird der Arzneimittelbegriff in § 2 Absatz 1 AMG wie folgt definiert:

„Arzneimittel […] sind Stoffe oder Zubereitungen aus Stoffen,

1. die zur Anwendung im oder am menschlichen Körper bestimmt sind und als Mittel mit Eigenschaften zur Heilung oder Linderung oder zur Verhütung menschlicher Krankheiten oder krankhafter Beschwerden bestimmt sind oder
2. die im oder am menschlichen Körper angewendet oder einem Menschen verabreicht werden können, um entweder
  1. die physiologischen Funktionen durch eine pharmakologische, immunologische oder metabolische Wirkung wiederherzustellen, zu korrigieren oder zu beeinflussen oder
  2. eine medizinische Diagnose zu erstellen.“

Danach erfolgt die Einstufung als Arzneimittel zum einen aufgrund der objektiven Zweckbestimmung, die u. a. durch Aufmachung, Kennzeichnung und Bewerbung geprägt ist (Nr. 1 – Präsentationsarzneimittel), und zum anderen aufgrund der Funktion (Nr. 2 – Funktionsarzneimittel). Trotz der folgenden allgemeinen Kriterien bleibt die Einstufung als Arzneimittel stets eine Einzelfallentscheidung.

#### Präsentationsarzneimittel

Bei der Prüfung eines potenziellen Präsentationsarzneimittels ist die überwiegende objektive Zweckbestimmung das maßgebliche Merkmal zur Abgrenzung. Definitionsgemäß dienen Nahrungsergänzungsmittel dem Zweck, die allgemeine Ernährung von gesunden Verbrauchern zu ergänzen. Hingegen sind Arzneimittel zur Heilung, Linderung oder Verhütung von Krankheiten oder krankhaften Beschwerden bestimmt. Die vom Inverkehrbringer angegebene Zweckbestimmung ist dabei nicht das alleinige Entscheidungsmerkmal, um ein Erzeugnis als Präsentationsarzneimittel einzustufen. Der Europäische Gerichtshof (EuGH) entschied, dass davon auszugehen ist, dass ein Erzeugnis nicht nur dann als „Mittel zur Heilung oder Verhütung von Krankheiten“ bezeichnet wird, wenn es ausdrücklich als solches bezeichnet wird, sondern auch dann, wenn bei einem durchschnittlich informierten Verbraucher auch nur schlüssig, aber mit Gewissheit der Eindruck entsteht, dass dieses Erzeugnis eine arzneiliche Wirkung haben müsse [10]. Es ist demnach vielmehr erforderlich, sich aus dem Zusammenspiel verschiedener Einzelhinweise auf ein Präsentationsarzneimittel eine wertende Gesamtbetrachtung zu erarbeiten. Zu berücksichtigen sind u. a. die stoffliche Zusammensetzung des Erzeugnisses, seine Darreichungsform und Verpackung, ebenso seine Bezeichnung, der Beipackzettel mit möglichen Hinweisen auf pharmazeutische Forschungen oder ärztliche Zeugnisse über bestimmte Eigenschaften sowie weitere dem Hersteller zurechenbare Informationen, Veröffentlichungen und Produktwerbung, die für den Verbraucher verfügbar sind [11]. An dieser Stelle ist jedoch zu betonen, dass nicht jede Angabe mit Krankheitsbezug zur Einstufung als Präsentationsarzneimittel führt. So gilt für Nahrungsergänzungsmittel beispielsweise das Verbot krankheitsbezogener Werbung nach Artikel 7 Absatz 3 Verordnung (EU) Nr. 1169/2011 [12], während zugelassene Angaben über die Reduzierung eines Krankheitsrisikos gemäß Artikel 14 Verordnung (EG) Nr. 1924/2006 [13] erlaubt sind. Unabhängig von der Verkehrsfähigkeit als Nahrungsergänzungsmittel ist die Möglichkeit einer Einstufung als Präsentationsarzneimittel nicht gegeben, sofern diese Angaben für das Gesamtbild der Produktpräsentation aus Sicht eines durchschnittlich informierten Verbrauchers nicht als prägend aufgefasst werden [14].

Es erscheint vielleicht verwunderlich, dass allein aufgrund einer bloßen Zweckbestimmung im rechtlichen Sinn ein Arzneimittel nach AMG vorliegt, obwohl das Erzeugnis selbst keinerlei arzneiliche Wirkung besitzt. Laut EuGH ist der Arzneimittelbegriff in dieser Form bewusst weit auszulegen, um Verbraucher nicht nur vor schädlichen oder giftigen Arzneimitteln zu schützen, sondern auch vor Erzeugnissen, die nicht ausreichend wirksam sind oder nicht wirksam, wie anhand der Bezeichnung erwartet werden darf, und welche dann anstelle geeigneter Heilmittel verwendet werden [15].

#### Funktionsarzneimittel

Für die Einstufung eines Erzeugnisses als Funktionsarzneimittel ist eine Gesamtbetrachtung aller Merkmale des Erzeugnisses zu berücksichtigen. Hierzu zählen insbesondere seine Zusammensetzung, seine pharmakologischen, immunologischen oder metabolischen Eigenschaften nach dem aktuellen Stand der Wissenschaft, die Modalitäten seines Gebrauchs, der Umfang seiner Verbreitung, seine Bekanntheit bei den Verbrauchern und die Risiken, die seine Verwendung mit sich bringen könnten [16]. Hierbei stellt die pharmakologische Wirkung das wesentliche Tatbestandsmerkmal für die Einstufung als Arzneimittel dar, welches aufgrund aktueller wissenschaftlicher Daten nachzuweisen ist [17, 18]. Es existiert für die pharmakologische Wirkung keine Legaldefinition, jedoch wird sie in einem Leitfaden der Medical Device Coordination Group, einer Expertengruppe der Europäischen Kommission zur Abgrenzung zwischen kosmetischen Mitteln und Arzneimitteln, wie folgt *wissenschaftlich* definiert:

„‚Pharmacological means’ is understood as an interaction typically at a molecular level between a substance or its metabolites and a constituent of the human body which results in initiation, enhancement, reduction or blockade of physiological functions or pathological processes […]“ [19].

Der pharmakologische Wirkmechanismus ist nach dieser Definition nicht nur bei Arzneimitteln zu finden, sondern auch bei Lebensmitteln des allgemeinen Verzehrs und Nahrungsergänzungsmitteln, die Stoffe mit ernährungsspezifischer oder physiologischer Wirkung enthalten. Daher müssen bei der Einstufung eines Erzeugnisses als Arzneimittel alle in § 2 Absatz 1 Nr. 2 a AMG genannten Kriterien in den Blick genommen werden. Nach der ständigen Rechtsprechung entfaltet ein Erzeugnis eine pharmakologische Wirkung, wenn es die menschlichen physiologischen Funktionen in *nennenswerter* Weise durch *gezielte* Steuerung wiederherstellt, korrigiert oder sonst positiv beeinflussen kann, wobei der bestimmungsgemäße, normale Gebrauch maßgeblich ist [20]. Hierbei sind unter den physiologischen Funktionen die normalen, im menschlichen Körper ablaufenden Lebensvorgänge zu verstehen. Aus der Notwendigkeit der Unterscheidung zu Wirkungen auf die physiologischen Funktionen von Lebensmitteln wurde von der Rechtsprechung der Begriff der sogenannten Erheblichkeitsschwelle entwickelt. Mit der hierbei vorausgesetzten *nennenswerten* Einwirkung auf die physiologischen Funktionen sollen Wirkungen ausgeschlossen werden, die einen Eingriff in Körperfunktionen darstellen, die völlig unerheblich sind und die physiologischen Funktionen nicht wirklich beeinflussen [21]. Das Kriterium einer gezielten, von außen gesteuerten Einwirkung dient zusätzlich dazu, eine Abgrenzung gegenüber der ernährungsspezifischen und physiologischen Wirkung vorzunehmen, also der Wirkung durch die unspezifische Nährstoffaufnahme durch Lebensmittel [22]. Dies einbeziehend, ist bei einer gezielten Zuführung von Nährstoffen die Erheblichkeitsschwelle dann überschritten und eine Wirkung als pharmakologisch anzusehen, wenn sie über die Wirkung hinausgeht, die durch den Verzehr eines Lebensmittels in angemessener Menge im menschlichen Körper ausgelöst werden kann [23].

Anders als bei Lebensmitteln, die die physiologischen Funktionen des Organismus lediglich aufrechterhalten, wird von der pharmakologischen Wirkung gefordert, dass sie diese wiederherstellt, korrigiert oder sonst positiv beeinflusst [24]. Nach der Auslegung des Bundesverwaltungsgerichts setzt die *Wiederherstellung* der physiologischen Funktionen voraus, dass die normalen Lebensvorgänge nicht mehr ordnungsgemäß ablaufen, genauso wie auch bei einer *Korrektur* von einer Abweichung vom normalen Funktionieren des Organismus ausgegangen wird. Das Kriterium der *Beeinflussung* der physiologischen Funktionen ist neben der Wiederherstellung und Korrektur als qualitativ gleichgestellt zu verstehen. So muss auch die Beeinflussung zu einer Veränderung führen, die außerhalb der normalen im menschlichen Körper ablaufenden Lebensvorgänge liegt [25].

Das Vorliegen einer pharmakologischen Wirkung ist darüber hinaus nicht an eine nachgewiesene therapeutische Wirksamkeit gebunden [26]. Ein Erzeugnis, das geeignet ist, therapeutische Zwecke zu erfüllen, ist im Wege des Erst-Recht-Schlusses in jedem Fall als ein Arzneimittel einzustufen [27]. Jedoch ist für eine Einstufung als Funktionsarzneimittel auch ein pharmakologischer Wirknachweis ohne therapeutische Anwendung ausreichend [28]. Diese Differenzierung kann dazu führen, dass ein Erzeugnis zwar keine therapeutische Wirksamkeit, wohl aber eine pharmakologische Wirkung aufweist. Das dann als Arzneimittel eingestufte Produkt bedarf für einen Marktzugang folglich eine Arzneimittelzulassung, welche es aufgrund der fehlenden therapeutischen Wirksamkeit jedoch gegebenenfalls nicht erlangen kann. Das Erzeugnis ist in diesem Fall weder als Lebensmittel noch als Arzneimittel verkehrsfähig, solange und soweit keine neuen Daten eine therapeutische Wirksamkeit belegen können [29].

Auch wenn die pharmakologische Wirkung und die Erheblichkeitsschwelle die wohl wichtigsten Merkmale zur Einstufung als Funktionsarzneimittel sind, sind auch die o. g. weiteren Kriterien der Gesamtbetrachtung bei der Einstufungsprüfung zu berücksichtigen. Insbesondere im Grenzbereich zwischen Nahrungsergänzungs- und Arzneimitteleigenschaften sind die möglichen Gesundheitsrisiken bei der Beurteilung mit einzubeziehen. Im Sinne des unionsrechtlichen Vorsorgegrundsatzes ist hier die Einstufung als Arzneimittel gerechtfertigt, sofern vernünftige Zweifel an der Unbedenklichkeit eines Erzeugnisses vorliegen [30].

### Sicherheit

Laut Artikel 14 Absatz 1 Verordnung (EG) Nr. 178/2002 dürfen Lebensmittel, die nicht sicher sind, nicht in Verkehr gebracht werden. Die Prüfung der Sicherheit liegt dabei in der Verantwortung des Lebensmittelunternehmers. Eine vorausgehende behördliche Zulassung oder Sicherheitsprüfung findet nicht statt. Vor dem Inverkehrbringen von Nahrungsergänzungsmitteln ist gemäß § 5 NemV lediglich eine Anzeige durch den Inverkehrbringer beim BVL erforderlich, die den zuständigen Behörden der Länder die stichprobenartige Überwachung der Einhaltung der lebensmittelrechtlichen Vorschriften erleichtern soll. Für Vitamine und Mineralstoffe gibt es bisher keine EU-weiten Höchstmengen. Für die Bewertung der Sicherheit von Lebensmitteln mit Mikronährstoffen können jedoch die Tolerable Upper Intake Levels (ULs) der Europäischen Behörde für Lebensmittelsicherheit (EFSA) herangezogen werden [31]. Darüber hinaus hat in Deutschland das Bundesinstitut für Risikobewertung Vorschläge für die Festlegung von Höchstmengen für Vitamine und Mineralstoffe erarbeitet [32].

Im Vergleich zu Lebensmitteln, die grundsätzlich sicher sein müssen, können Arzneimittel, die mit dem Ziel der Heilung, Linderung oder Verhütung von Krankheiten eingesetzt werden, unter Umständen auch unerwünschte Arzneimittelwirkungen zeigen. Im Zuge der verpflichtenden Arzneimittelzulassung gemäß § 21 AMG müssen Hersteller neben der Wirksamkeit auch die Unbedenklichkeit und pharmazeutische Qualität belegen. Sofern die Nebenwirkungen die erwünschten Effekte überwiegen, also ein ungünstiges Nutzen-Risiko-Verhältnis feststeht, wird nach § 25 Absatz 2 Nr. 5 AMG die Zulassung nicht erteilt.

### Gemeinsame Expertenkommission

Zuständig für die Einstufung von Erzeugnissen als Arzneimittel sind die für die Arzneimittelüberwachung zuständigen Behörden der (Bundes‑)Länder, gegebenenfalls nach § 21 Absatz 4 AMG in Zusammenarbeit mit dem Bundesinstitut für Arzneimittel und Medizinprodukte (BfArM). Bei Borderline-Produkten ist die Abgrenzung häufig sehr aufwendig, da in vielen Fällen wissenschaftliche Daten oder eine fundierte gesundheitliche Risikobewertung fehlen. Die Gemeinsame Expertenkommission zur Einstufung von Stoffen des BVL und des BfArM erarbeitet Stellungnahmen, um diese Lücke zu schließen. Sie ist ein interdisziplinäres, unabhängiges Gremium, das sich mit Einstufungs- und Abgrenzungsfragen befasst. Die Stellungnahmen der Gemeinsamen Expertenkommission sind zwar rechtlich nicht bindend, haben jedoch als Sachverständigengutachten einen bedeutenden Nutzen für die Überwachungsbehörden und Industrievertreter. Die Kommission hat u. a. ein Grundsatzpapier zur pharmakologischen Wirkung veröffentlicht, in dem die naturwissenschaftliche und juristische Auslegung des Begriffs sowie weitere Vorgaben zur Einstufung von Funktionsarzneimitteln zusammengetragen wurden. Darüber hinaus hat das Gremium bereits umfangreiche Stellungnahmen zu den Mikronährstoffen Vitamin D, Selen und Zink erarbeitet. Alle drei Nährstoffe werden sowohl in Nahrungsergänzungsmitteln als auch in Arzneimitteln eingesetzt, wobei sich die Dosierungen beider Produktkategorien überschneiden und somit oftmals die Frage der Abgrenzung gestellt wird. Anhand der folgenden stark verkürzten Stellungnahme der Gemeinsamen Expertenkommission zu zinkhaltigen Produkten wird deutlich, wie zum einen das allgemeine Vorgehen auf konkrete Nährstoffe anzuwenden ist und gleichzeitig die einzelfallbezogenen Faktoren des jeweiligen Nährstoffs berücksichtigt werden müssen [33].

Der Mineralstoff Zink findet sich sowohl in Nahrungsergänzungsmitteln häufig in einer Dosierung zwischen 5 mg und 50 mg pro Tag als auch in Arzneimitteln mit vergleichbarer Dosierung von 10 mg bis 250 mg pro Tag. Zur Abgrenzung und Prüfung auf das Vorliegen eines Funktionsarzneimittels ist die geringste Dosis einer belegten pharmakologischen Wirkung zu bestimmen. Hierfür führt die Gemeinsame Expertenkommission die Aufbereitungsmonographie Zink des Bundesgesundheitsamtes (1994) auf, wonach eine orale Applikation von 10 mg Zink pro Tag bei einem nachgewiesenen Zinkmangel, z. B. Akrodermatitis, zur Therapie bei Morbus Wilson und zur Therapie mit Penicillamin therapeutisch angewandt wird [34]. Durch die nachgewiesene therapeutische Wirksamkeit ist für eine Tagesdosierung von 10 mg Zink erst recht eine pharmakologische Wirkung im Sinne eines Funktionsarzneimittels belegt.

Zusätzlich zu einer pharmakologischen Wirkung muss jedoch auch die Erheblichkeitsschwelle überschritten werden. Wenn eine infrage stehende Zinkmenge auch über den Verzehr von Lebensmitteln in angemessener Menge aufgenommen werden kann, erfüllt diese Dosierung lediglich eine Aufrechterhaltung der physiologischen Funktionen und Nahrungsergänzungsmittel mit dieser Tagesverzehrsmenge können nicht als Funktionsarzneimittel eingestuft werden. Laut der Nationalen Verzehrstudie II (NVS II) liegt die Zinkaufnahme im Median im 95. Perzentil bei 20,2 mg pro Tag für Männer und 15,1 mg pro Tag für Frauen [35]. Es ist darüber hinaus plausibel, dass auch höhere Zinkmengen über die allgemeine Ernährung aufgenommen werden. Längerfristig kann eine zu hohe Zinkaufnahme zu gesundheitlich unerwünschten Wirkungen führen. Das Scientific Committee on Food (SCF) hat hierfür einen UL von 25 mg Zink pro Tag festgelegt [36]. Der UL kann jedoch im Rahmen eines angemessenen Verzehrs von allgemeinen Lebensmitteln, insbesondere bei der Auswahl von Lebensmitteln mit hohem Zinkgehalt, erreicht oder sogar überschritten werden. Aufgrund der hohen Exposition über Lebensmittel des allgemeinen Verzehrs liegt die Erheblichkeitsschwelle bereits im Bereich oberhalb des UL und ist damit nicht weiter zu konkretisieren. Schlussendlich liegt bei Erzeugnissen mit einer empfohlenen Tagesdosis von über 25 mg Zink ein nicht sicheres Lebensmittel gemäß Artikel 14 Verordnung (EG) Nr. 178/2002 vor. Für die praktische Beurteilung der Verkehrsfähigkeit kann die Anwendbarkeit der Erheblichkeitsschwelle bzw. die Festlegung, ab welcher Dosierung von einem Funktionsarzneimittel auszugehen wäre, daher offenbleiben.

### Arznei- und Lebensmittelüberwachung

Neben der inhaltlichen Auseinandersetzung zur Abgrenzung zwischen Nahrungsergänzungsmitteln und Arzneimitteln wird die amtliche Lebensmittel- und Arzneimittelüberwachung vor weitere Herausforderungen gestellt. So liegt die Zuständigkeit der Überwachung im föderalen System der Bundesrepublik beim jeweils zuständigen Bundesland. Obwohl das grundsätzliche Vorgehen bei der Abgrenzung identisch ist, können die Überwachungsbehörden der Bundesländer bei der Ausübung ihres Ermessens zu unterschiedlichen Ergebnissen kommen. Da ein einheitlicher Vollzug angestrebt wird, stimmen sich die Bundesländer u. a. in Gremien wie dem Arbeitskreis Lebensmittelchemischer Sachverständiger der Länder und des Bundesamtes für Verbraucherschutz und Lebensmittelsicherheit (ALS) und der Arbeitsgruppe Arzneimittel‑, Apotheken‑, Transfusions- und Betäubungsmittelwesen (AG AATB) ab. Darüber hinaus unterstützen verschiedene Einrichtungen des Bundes die Überwachungsbehörden, z. B. durch die Stellungnahmen der Gemeinsamen Expertenkommission.

Aufgrund der sich überschneidenden Abgrenzungsprüfung besteht zwischen der Lebensmittel- und Arzneimittelüberwachung ein hoher Abstimmungsbedarf. Soweit die Prüfung eines Erzeugnisses durch die zuständige Lebensmittelüberwachungsbehörde eine Einstufung als Arzneimittel ergibt, wird der Fall sowie dessen weitere Verfolgung entsprechend der weiteren Zuständigkeit an die Arzneimittelüberwachungsbehörde abgegeben. Ein entsprechendes Vorgehen erfolgt im Umkehrschluss durch die Arzneimittelüberwachung, soweit deren Prüfung das Vorliegen eines Lebensmittels bejaht.

### Kontrolle des Online-Handels

Immer mehr Menschen kaufen Arznei- und Nahrungsergänzungsmittel über das Internet. So gaben 21 % der Befragten im Alter von 16 bis 74 Jahren an, im Jahr 2024 Arzneimittel oder Nahrungsergänzungsmittel online gekauft zu haben.^1^ Während die Probenahme im stationären Handel seit Jahrzehnten routiniert durchgeführt wird, birgt die Überwachung des Online-Handels einige Herausforderungen. Um die Lebensmittelüberwachungsbehörden bei dieser Aufgabe zu unterstützen, wurde im Juli 2013 die gemeinsame Zentralstelle „Kontrolle der im Internet gehandelten Erzeugnisse des LFGB und Tabakerzeugnisse“ (G@ZIELT) ins Leben gerufen. Verbraucher sollen ein gleich hohes Niveau an Lebensmittelsicherheit beim Einkauf im Internet wie im stationären Handel erwarten können. Dafür ist G@ZIELT in der Lage, Proben auch aus dem Internet zu beziehen, und steht mit diversen Online-Marktplätzen im engen Kontakt, um gegen lebensmittelrechtliche Verstöße vorgehen zu können. Für interessierte Kreise veröffentlicht G@ZIELT jährlich einen Bericht über Kontrollprogramme sowie Ergebnisse.^2^

### Harmonisierung in der Europäischen Union

Trotz der harmonisierten rechtlichen Grundlagen erfolgt die Abgrenzungspraxis auch auf europäischer Ebene nicht gänzlich einheitlich. So ist es möglich und nach dem aktuellen Stand des Gemeinschaftsrechts auch zulässig, dass die Mitgliedsstaaten bei der Bewertung der vorliegenden Nachweise für ein Funktionsarzneimittel zu unterschiedlichen Ergebnissen kommen [37]. So kann ein Erzeugnis unter Umständen in einem Mitgliedstaat rechtmäßig als Lebensmittel/Nahrungsergänzungsmittel in Verkehr sein und gleichzeitig in einem anderen Mitgliedstaat unter das nationale Arzneimittelrecht fallen.

Ein nächster Schritt in Richtung Harmonisierung stellt die Festlegung von in der Europäischen Union einheitlichen Höchstmengen für Vitamine und Mineralstoffe in Lebensmitteln dar. Die Mitgliedstaaten tauschen sich kontinuierlich in den Sitzungen der Kommissionsarbeitsgruppe „Working Group on Food Supplements and Fortified Foods“ zu einem einheitlichen Vorgehen und zu aktuellen Themen aus.

## Fazit

Die Abgrenzung zwischen Nahrungsergänzungsmitteln und Arzneimitteln stellt sowohl regulatorisch als auch praktisch eine Herausforderung dar. Zwar existieren durch die Rechtsprechung bereits einige Werkzeuge zur Einstufung solcher Produkte, dennoch handelt es sich stets um Einzelfallentscheidungen, bei denen verschiedene Faktoren – etwa die pharmakologische Wirkung, die Erheblichkeitsschwelle und die Aufmachung – zu beachten sind.

Neben der inhaltlichen Bewertung von Borderline-Produkten ist eine Auseinandersetzung mit der verwaltungsrechtlichen Struktur und der aktuellen Marktsituation unerlässlich. Die föderale Zuständigkeitsverteilung zwischen den Bundesländern sowie zwischen den jeweiligen Lebensmittel- und Arzneimittelüberwachungsbehörden erfordert ein hohes Maß an Koordination. Zusätzlich sehen sich die Überwachungsbehörden mit einem wachsenden Online-Handel konfrontiert, dessen Dynamik und Umfang eine flächendeckende Kontrolle erschweren.

Trotz dieser Herausforderungen funktioniert das System in der Praxis: Die Überwachungsbehörden nehmen qualifizierte und rechtssichere Einstufungen neuer Produkte vor. Unterstützt werden sie dabei durch verschiedene Gremien, Arbeitsgruppen und Einrichtungen auf Bundes- und Landesebene, etwa die Gemeinsame Expertenkommission sowie die Arbeitsgruppen des ALS und AATB. Gleichwohl wird es in einem sich wandelnden und heterogenen Produktumfeld wie dem der Nahrungsergänzungsmittel und Arzneimittel auch künftig zu unterschiedlichen Auffassungen in Abgrenzungsfragen kommen – diese sind Ausdruck der Komplexität und Dynamik des Marktes.

## Footnotes

1 https://www.destatis.de/DE/Presse/Pressemitteilungen/Zahl-der-Woche/2025/PD25_11_p002.html#%3A%7E%3Atext%3DWie%2520das%2520Statistische%2520Bundesamt%2520%28Destatis%2Cer%2520noch%2520bei%252016%2520%2525%2520gelegen. Zugegriffen: 23.05.2025.

2 https://www.bvl.bund.de/DE/Arbeitsbereiche/01_Lebensmittel/04_AntragstellerUnternehmen/11_G@ZIELT_Onlinehandel/lm_ueberwachung_internethandel_node.html. Zugegriffen: 23.05.2025.

## References

[1] Nahrungsergänzungsmittelverordnung vom 24. Mai 2004 (BGBl. I S. 1011), die zuletzt durch Artikel 11 der Verordnung vom 5. Juli 2017 (BGBl. I S. 2272) geändert worden ist

[2] Bundesamt für Verbraucherschutz und Lebensmittelsicherheit (2025) Anzahl der Anzeigen nach § 5 der Nahrungsergänzungsmittelverordnung (NemV). https://www.bvl.bund.de/SharedDocs/Downloads/01_Lebensmittel/nem/Anzah_Anzeigen_nach_%C2%A7_5_der_NemV.pdf?__blob=publicationFile&v=3. Zugegriffen: 23. Mai 2025

[3] Bechthold A, Albrecht V, Leschik-Bonnet E, Heseker H (2012) DGE-Stellungnahme: Beurteilung der Vitaminversorgung in Deutschland Teil 1: Daten zur Vitaminzufuhr. Ernahr Umsch. 10.4455/eu.2012.974

[4] Max Rubner-Institut (2013) Nationale Verzehrsstudie II. Lebensmittelverzehr und Nährstoffzufuhr auf Basis von 24 h-Recalls. https://www.mri.bund.de/fileadmin/MRI/Institute/EV/Lebensmittelverzehr_N%C3%A4hrstoffzufuhr_24h-recalls-neu.pdf. Zugegriffen: 23. Mai 2025

[5] Verordnung (EG) Nr. 178/2002 des Europäischen Parlaments und des Rates vom 28. Januar 2002 zur Festlegung der allgemeinen Grundsätze und Anforderungen des Lebensmittelrechts, zur Errichtung der Europäischen Behörde für Lebensmittelsicherheit und zur Festlegung von Verfahren zur Lebensmittelsicherheit (ABl. L 031 vom 01.02.2002, S. 1)

[6] Arzneimittelgesetz in der Fassung der Bekanntmachung vom 12. Dezember 2005 (BGBl. I S. 3394), das zuletzt durch Artikel 2 des Gesetzes vom 23. Oktober 2024 (BGBl. 2024 I Nr. 324) geändert worden ist

[7] BVerwG, Urteil vom 07.11.2019, 3 C 19/18, Rn. 14 – juris

[8] EuGH, Urteil vom 15.01.2009, C‑140/07, Rn. 23 ff. – juris (Hecht Pharma); BVerwG, Urteil vom 26.05.2009, 3 C 5.09, Rn. 15 – juris (Red Rice)

[9] Richtlinie 2001/83/EG des Europäischen Parlaments und des Rates vom 6. November 2001 zur Schaffung eines Gemeinschaftskodexes für Humanarzneimittel (ABl. L 311 vom 28.11.2001, S. 67)

[10] EuGH, Urteil vom 30.11.1983, C‑222/82, Rn. 18 – juris (van Bennekom)

[11] OVG NRW, Urteil vom 26.09.2019, 13 A 3290/17, Rn. 56 – juris

[12] Verordnung (EU) Nr. 1169/2011 des Europäischen Parlaments und des Rates vom 25. Oktober 2011 betreffend die Information der Verbraucher über Lebensmittel und zur Änderung der Verordnungen (EG) Nr. 1924/2006 und (EG) Nr. 1925/2006 des Europäischen Parlaments und des Rates und zur Aufhebung der Richtlinie 87/250/EWG der Kommission, der Richtlinie 90/496/EWG des Rates, der Richtlinie 1999/10/EG der Kommission, der Richtlinie 2000/13/EG des Europäischen Parlaments und des Rates, der Richtlinien 2002/67/EG und 2008/5/EG der Kommission und der Verordnung (EG) Nr. 608/2004 der Kommission (ABl. L 304 vom 22.11.2011, S. 18)

[13] Verordnung (EG) Nr. 1924/2006 des Europäischen Parlaments und des Rates vom 20. Dezember 2006 über nährwert- und gesundheitsbezogene Angaben über Lebensmittel (ABl. L 404 vom 30.12.2006, S. 9)

[14] OVG Lüneburg, Urteil vom 03.02.2011, 13 LC 92/09, Rn. 14 – juris

[15] EuGH, Urteil vom 30.11.1983, C‑227/82, Rn. 17 – juris (van Bennekom)

[16] EuGH, Urteil vom 15.11.2007, C‑319/05, Rn. 55 – juris (Kommission/BRD); EuGH, Urteil vom 09.06.2005, C 211/03, Rn. 51 – juris (HLH Warenvertrieb und Orthica)

[17] EuGH, Urteil vom 15.11.2007, C‑319/05, Rn. 59 – juris (Kommission/BRD EuGH); Urteil vom 09.06.2005, C 211/03, Rn. 52 – juris (HLH Warenvertrieb und Orthica); BVerwG, Urteil vom 26.05.2009, 3 C 5.09, Rn. 13, 15 – juris (Red Rice)

[18] EuGH, Urteil vom 15.01.2009, C‑140/07, Rn. 26 – juris (Hecht Pharma)

[19] Medical Device Coordination Group (2024) Guidance on borderline between medical devices and medicinal products under Regulation (EU) 2017/745 on medical devices. MDCG 2022‑5 rev.1. https://health.ec.europa.eu/latest-updates/mdcg-2022-5-rev1-guidance-borderline-between-medical-devices-and-medicinal-products-under-regulation-2024-10-29_en. Zugegriffen: 23. Mai 2025

[20] EuGH, Urteil vom 06.09.2012, C 308/11, Rn. 35 – juris (Chemische Fabrik Kreussler); EuGH, Urteil vom 30.04.2009, C‑27/08, Rn. 21 ff. – juris (Weihrauch H 15-Tabletten); BVerwG, Urteil vom 20.11.2014, 3 C 27.13, Rn. 24f. – juris (E-Zigarette); BVerwG, Urteil vom 26.05.2009, 3 C 5.09, Rn. 15 – juris (Red Rice), jeweils m. w. N.

[21] EuGH, Urteil vom 16.04.1991, C‑112/89, Rn. 22 – juris (Upjohn); BVerwG, Urteil vom 16.05.2007, 3 C 34.06, Rn. 29 – juris (Kampherhaltige Pferdesalbe)

[22] BVerwG, Urteil vom 14.12.2006, 3 C 40/05, Rn. 22 – juris; OVG Lüneburg, Urteil vom 29.09.2021, 13 LB 31/14, Rn. 77 – juris (Ginkgo)

[23] EuGH, Urteil vom 15.11.2007, C 319/05, Rn. 68 – juris (Kommission/BRD); BVerwG, Urteil vom 25.07.2007, 3 C 21.06, Rn. 29 – juris (OPC); BVerwG, Urteil vom 14.12.2006, 3 C 40.05, Rn. 22 – juris; BGH, Urteil vom 26.06.2008, I ZR 112/05, Rn. 21 – juris (HMB-Kapseln); BGH, Urteil vom 11.07.2002, I ZR 34/01, Rn. 67 – juris

[24] EuGH, Urteil vom 15.01.2009, C‑140/07, Rn. 42 –juris (Hecht Pharma); EuGH, Urteil vom 30.04.2009, C‑27/08, Rn. 23 ff. – juris (BIOS Naturprodukte); EuGH, Urteil vom 06.09.2012, C 308/11, Rn. 35 – juris (Chemische Fabrik Kreussler); BVerwG, Urteil vom 26.05.2009, 3 C 5.09, Rn. 15 – juris (Red Rice)

[25] BVerwG, Urteil vom 25.07.2007, 3 C 21.06, Rn. 28 – juris (OPC)

[26] BVerwG, Urteil vom 25.07.2007, 3 C 21.06 Rn. 26 – juris (OPC)

[27] BVerwG, Urteil vom 25.07.2007, Az. 3 C 21.06, Rn. 26 – juris (OPC)

[28] BVerwG, Urteil vom 14.12.2006, Az. 3 C 40.05, Rn. 23 – juris (Tibetische Kräutermischung)

[29] OVG Lüneburg, Urteil vom 29.09.2021, 13 LB 31/14, Rn. 79 – juris (Ginkgo); BVerwG, Vorlagebeschluss vom 14.12.2006, 3 C 38.06, Rn. 31 – juris (Red Rice)

[30] BVerwG, Urteil vom 07.11.2019, 3 C 19.18, Rn. 31 ff. m. w. N. – juris

[31] European Food Safety Authority (2024) Overview on Tolerable Upper Intake Levels as derived by the Scientific Committee on Food (SCF) and the EFSA Panel on Dietetic Products, Nutrition and Allergies (NDA). https://www.efsa.europa.eu/sites/default/files/2024-05/ul-summary-report.pdf. Zugegriffen: 8. Juli 2025

[32] Bundesinstitut für Risikobewertung (2024) Aktualisierte* Höchstmengenvorschläge für Vitamine und Mineralstoffe in Nahrungsergänzungsmitteln und angereicherten Lebensmitteln

[33] Gemeinsame Expertenkommission zur Einstufung von Stoffen (2023) Stellungnahme zur Einstufung von zinkhaltigen Produkten (Nr. 01/2023). https://www.bvl.bund.de/SharedDocs/Downloads/01_Lebensmittel/expertenkommission/Stellungnahme_zinkhaltigen_Produkte.pdf;jsessionid=555E4286E29423A4C96C8A8989B0DDF5.internet982?__blob=publicationFile&v=2. Zugegriffen: 23. Mai 2025

[34] BGA (1994) Aufbereitungsmonographie Zink. Bundesanzeiger Nr. 39 vom 25. Febr. 1994

[35] Max Rubner-Institut (2008) Ernährungsbericht Teil 2. Nationale Verzehrsstudie II. https://www.mri.bund.de/fileadmin/MRI/Institute/EV/NVSII_Abschlussbericht_Teil_2.pdf. Zugegriffen: 23. Mai 2025

[36] Scientific Committee on Food (2003) Opinion of the scientific committee on food on the tolerable upper intake level of zinc. https://ec.europa.eu/food/fs/sc/scf/out177_en.pdf. Zugegriffen: 23. Mai 2025

[37] EuGH, Urteil vom 15.01.2009, C‑140/07, Rn. 28 – juris (Hecht-Pharma); EuGH, Urteil vom 09.06.2005, C‑211/03 Rn. 56 – juris (HLH Warenvertrieb und Orthica)

